# Effectiveness of alternative semester break schedules on reducing COVID-19 incidence on college campuses

**DOI:** 10.1038/s41598-022-06260-1

**Published:** 2022-02-08

**Authors:** Chris L. Lehnig, Eyal Oren, Naveen K. Vaidya

**Affiliations:** 1 Computational Science Research Center, San Diego State University, San Diego, USA; 2 Division of Epidemiology and Biostatistics, School of Public Health, San Diego State University, San Diego, USA; 3 Department of Mathematics and Statistics, San Diego State University, San Diego, USA; 4 Viral Information Institute, San Diego State University, San Diego, USA

**Keywords:** Infectious diseases, Viral infection, Applied mathematics, Computational science, Epidemiology

## Abstract

Despite COVID-19 vaccination programs, the threat of new SARS-CoV-2 strains and continuing pockets of transmission persists. While many U.S. universities replaced their traditional nine-day spring 2021 break with multiple breaks of shorter duration, the effects these schedules have on reducing COVID-19 incidence remains unclear. The main objective of this study is to quantify the impact of alternative break schedules on cumulative COVID-19 incidence on university campuses. Using student mobility data and Monte Carlo simulations of returning infectious student size, we developed a compartmental susceptible-exposed-infectious-asymptomatic-recovered (SEIAR) model to simulate transmission dynamics among university students. As a case study, four alternative spring break schedules were derived from a sample of universities and evaluated. Across alternative multi-break schedules, the median percent reduction of total semester COVID-19 incidence, relative to a traditional nine-day break, ranged from 2 to 4% (for 2% travel destination prevalence) and 8–16% (for 10% travel destination prevalence). The maximum percent reduction from an alternate break schedule was estimated to be 37.6%. Simulation results show that adjusting academic calendars to limit student travel can reduce disease burden. Insights gleaned from our simulations could inform policies regarding appropriate planning of schedules for upcoming semesters upon returning to in-person teaching modalities.

## Introduction

The return of students to college campuses after scheduled breaks has resulted in dramatic surges of COVID-19 cases in college communities and has been linked to increases in county-level SARS-CoV-2 incidence^1,2^. Undocumented infection found significantly among college students has also played an important role in disseminating disease in the past year^3^. In response, many U.S. universities replaced their traditional weeklong spring break with multiple breaks of shorter durations in spring 2021. The relative effect alternative break schedules might have on COVID-19 incidence, however, remains uncertain. Despite the development of vaccines, due to the difficulty of local-level implementation, the uncertainty about length of immunity due to vaccine, public hesitancy of vaccine, and the emergence of SARS-CoV-2 variants, the decision on a proper academic schedule is critical to prevent surges in campus communities. Modeling SARS-CoV-2 transmission influenced by student travel and disease prevalence at travel destinations can provide insights into the impact different break schedules have on COVID-19 incidence among on-campus university students^4^.

Given the impact of COVID-19 on human mobility patterns and vice versa, others have examined how changes in mobility might impact spread of SARS-CoV-2. Since mobility can represent proxies for social distancing and stay-at-home interventions, these data sources may present approaches to examining impacts on disease transmission. For example, mobility networks derived from cell phone data predict that a small minority of ‘superspreader’ points of interest account for a large majority of infections^5^. Similarly, studies have used Apple mobility data to examine financial markets^6^, and Google mobility data from 130 countries to show that even slight reductions in visits to various locations results in fewer COVID-19 cases and deaths^7^. In another country-level analysis, using both Apple and Google mobility data, mobility explained a substantial proportion of COVID-19 transmission variability. Therefore, consideration of mobility patterns, including those of students during semester breaks, is critical for modeling COVID-19 incidences on college campuses^8^^.^

While a few modeling studies have provided some insights into COVID-19 spread in college campuses^9–14^, none of the previous studies has explored the inter-relation among break schedule, travel behavior, and local COVID-19 surge. In this paper, we used mathematical and computational models to examine the effects of four commonly practiced alternative spring break schedules under five different realistic mean prevalence rates at travel destinations, on SARS-CoV-2 transmission among on-campus students (those not involved in virtual learning who physically go to campus). In particular, break schedules were derived from a sample of twenty-five universities that implemented altered spring break schedules in the spring 2021 semester. Analysis of mobility data, paired with results from Monte Carlo (MC) simulations, informed the relationship between travel and infections and were used directly in a variant of a SEIAR (susceptible-exposed-infectious-asymptomatic-recovered) type compartmental model to estimate total cases throughout the semester. We utilized both real-time and synthetic data to model the effects of breaks of different duration on college campuses. Furthermore, we performed thorough sensitivity analyses on those parameters that drive the results.

## Results

### Alternate break schedules

We analyzed the data from twenty-five sampled U.S. universities to observe the different break schedules, i.e., number of breaks and timing of the breaks, implemented in spring 2021. Our analysis led to the following four alternative spring break schedules: (1) the one-break schedule (nine-day break starting on day 72 of the semester), (2) the two-break schedule (a five- and four-day break on days 39 and 88 of the semester, respectively), (3) the three-break schedule (a four-, three-, and two-day break on days 27, 54, and 81, respectively), and (4) the four-break schedule (a three-, three-, two-, and one-day break on days 31, 52, 73, and 85, respectively). Note that each break considered was of the total length of nine days, which allowed us to perform a fair comparison among them.

### Infection among returning students after travel during semester break

**Figure 1:**
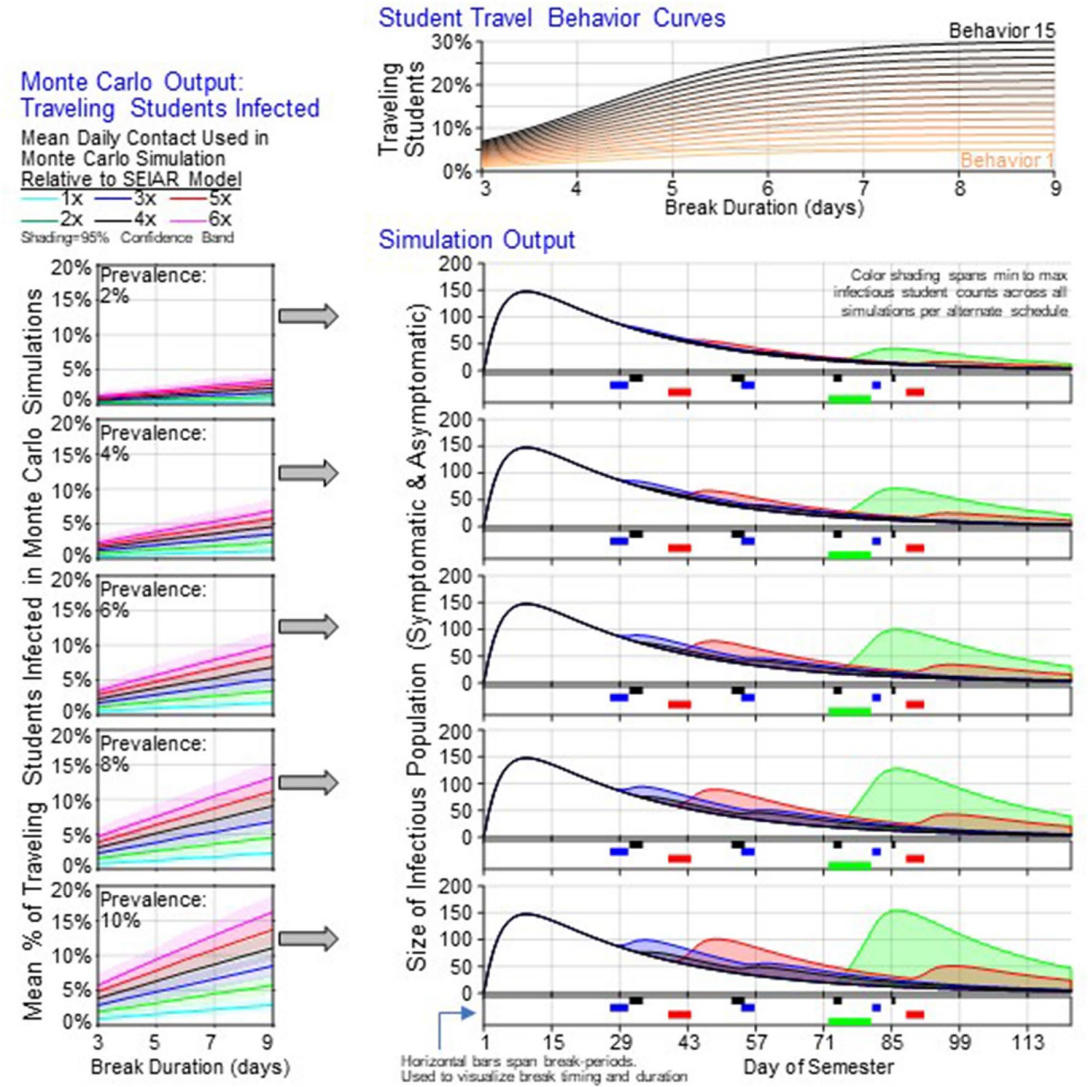
Break-period dependent information fed into SEIAR simulation and resulting range of infectious population size over time across all scenarios. The Monte Carlo Output panels show results of MC simulations. The prevalence rate in each panel provides the rate used in the simulation. Lines are colored by mean daily contact rate used in simulation. Plotted data are mean percent of infectious students. Colored bands show the 95% range of means across 500 simulations. Student Travel Behavior Curves show the relationship between break-duration and percent of students that travel used in simulations. Simulation Output shows the range of infectious student population sizes per alternative schedule (green: one-break, red: two-break, blue: three-break. black: four-break), across all combinations of student travel behavior curves and mean daily contacts while traveling parameters.

We performed MC simulations to identify the expected number of students who returned infected after each break. MC simulation results of infected percentage of returned students for various travel duration (three-day to nine-day break), various numbers of contacts during the break (10 daily contacts to 60 daily contacts), and various travel destination prevalence (2% to 10% destination prevalence) are summarized in Fig. 1 (bottom left). We observed that for a given mean daily contact and destination prevalence rate, the relationship between break-duration and percent of students who returned infected was approximately linear with positive correlation. In the 2% destination prevalence scenario, percent of returning students infected ranged from 0.21% (three-day break, 10 daily contacts) to 3.5% (nine-day break, 60 daily contacts). Similarly, this range increased to 0.42–6.9%, 0.6–10.1%, 0.8–13.2%, and 0.9–16.3% at a destination prevalence of 4%, 6%, 8%, and 10%, respectively.

### Temporal epidemic pattern among students over a semester

For each alternate break schedule identified, we used our transmission dynamics model to predict the temporal pattern of infectious student population for the entire semester (Fig. 1, bottom right). As expected, we observed that the longest nine-day break resulted in a large surge following the break (Green line, Fig. 1, bottom right). The peak of the surge increased as student behavior led to higher contact during travel or the travel prevalence at the destination increased. Importantly, our model predicts that the single surge was distributed into many surges of lower scale when multiple breaks were implemented. The larger the number of breaks, the smaller the surge size. As a consequence, in the largest number of breaks, i.e., the four-break schedule considered in this study, the surge was virtually negligible (Black curve, Fig. 1, bottom right).

### Cumulative COVID-19 cases in student body over a semester

**Figure 2:**
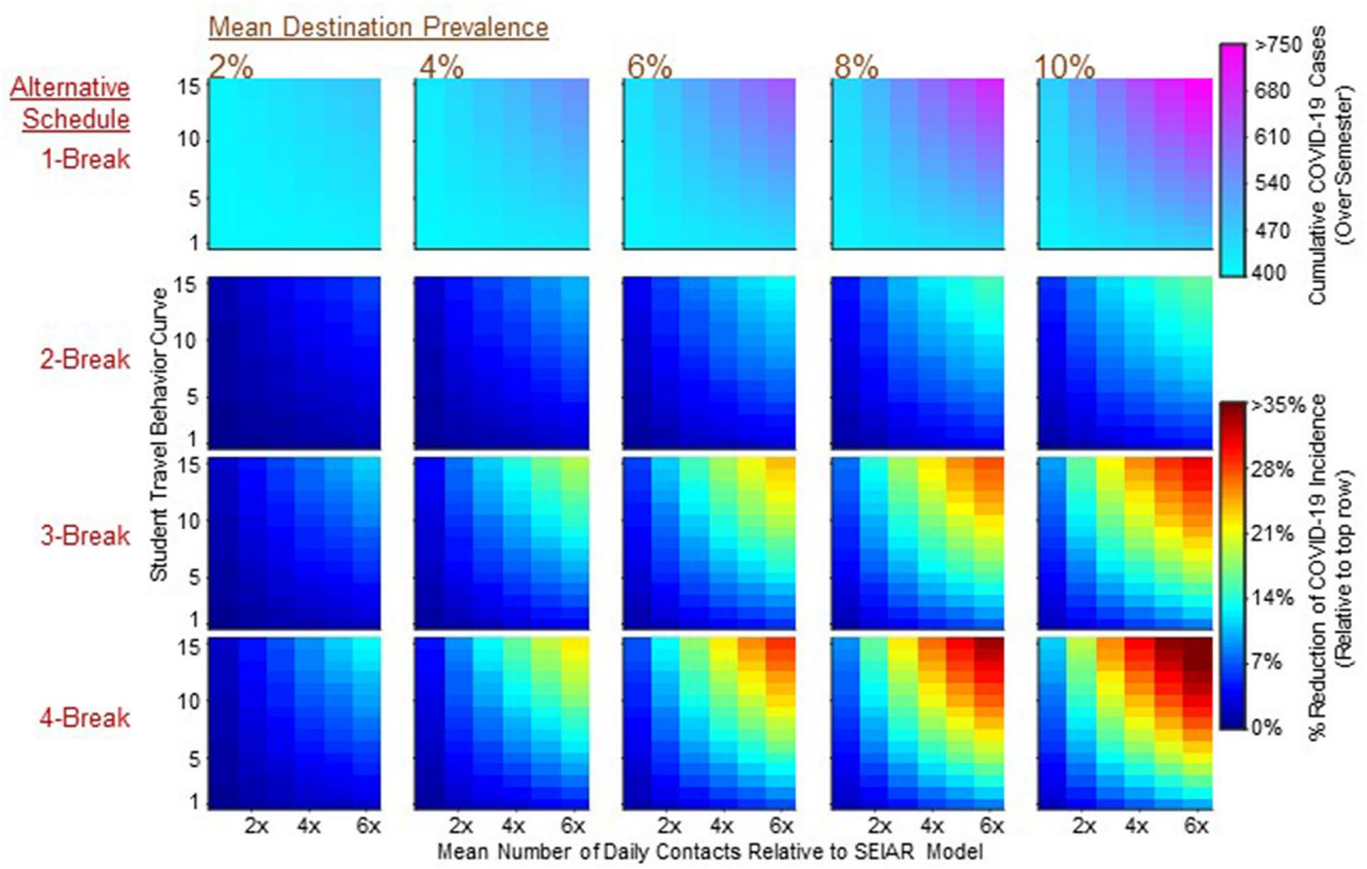
Sensitivity of the reduction of total cases for each break schedule (1800 simulations). The top row shows total COVID-19 incidence from the one-break schedule among on-campus students over the course of the semester. Remaining rows show the percent reduction in total incidence, relative to the one-break schedule, resulting from implementing two-, three- and four-break schedules. Within each panel, Y axis ticks correspond to the student travel behavior curve (how many students travel) and X axis ticks correspond to the mean daily contract rate used in the MC simulation (how many traveling students were infected). Each column corresponds to a mean travel destination prevalence rate. Each cell, within each panel, corresponds to one simulation.

For all schedule-prevalence scenarios, sensitivity of total COVID-19 cases to the number of students that travel and the proportion of them that return infected (identified by MC simulations) is shown in Fig. 2. Our results show that, under the one-break schedule, 407 to 751 students (out of 5,000) were infected with COVID-19 during the semester. Relative to the one-break schedule, the two-break schedule resulted in the reduction of total infections by 0.2% to 6.4% in the 2% destination prevalence scenario and by 0.1% to 16% in the 10% destination prevalence scenario. The three-break schedule was a considerable improvement over the two-break schedule, reducing the one-break schedule cases by a minimum of 0.39% (2% destination prevalence) to a maximum of 31% (10% destination prevalence). The most distributed schedule, the four-break schedule, showed similar outcomes to the three-break schedule but with a pronounced improvement, particularly in scenarios with a larger number of traveling students and a higher daily contact (upper left region of each panel in Fig. 2). In this scenario, cases were reduced by a minimum of 0.37% (2% destination prevalence) to a maximum of 37.6% (10% destination prevalence) relative to the one-break schedule. In general, the median percent reduction of total case from the one-break schedule is 2.1% to 7.6% in the two-break schedule, 3.6% to 13.9% in the three-break schedule, and 4.2% to 16.5% in the four-break schedule for low to high destination prevalence rates, respectively.

Regardless of schedule-prevalence pair and the number of students that travel, total cases and the percent reduction of total cases was always smallest under the scenarios where students maintained their contact rates while traveling at the same level as the during-school contact rates (Fig. 2).

## Discussion

The threat of COVID-19 transmission has been even greater because of the emergence of novel viral strains such as lineages B.1.1.529 (Omicron variant), B.1.617.2 (Delta variant) and B.1.1.7 (Alpha variant)^15^. In addition, concerns among students regarding vaccine safety and efficacy may create obstacles to achieving herd or sufficient population immunity at US university campuses^16^. Given current uncertainty on various aspects of COVID-19, it is critical to evaluate the effects of the semester break schedule on a COVID-19 surge among the student body and to identify whether any alternate schedules can be beneficial to avoid surges. Here, we developed a computational and mathematical model to study four alternate schedules that were implemented in some US universities during spring 2021.

Our results over a wide range of parameter space show that the alternative spring break, i.e., multiple breaks of a shorter length, can significantly reduce semester-wide cumulative COVID-19 cases among college campus students. This was achieved by distributing the large surge due to the traditional longest one-break into multiple surges of significantly small size. We also note that the estimated effectiveness of alternative spring break schedules on COVID-19 case reduction was very much dependent on student movement/contact dynamics and travel destination prevalence rates. This is consistent with work showing the role of mobility reduction in reducing infections^5^. This result is in line with modeling estimates implying that counties with more early spring break students had a higher growth rate of cases than counties with fewer early spring break students in 2020^17^.

If a university has reason to believe that many of its students will not heed public health warnings and travel for recreation during scheduled break periods, the university may want to consider implementing alternative break schedules, along with other mitigation measures such as vaccination, masking, and surveillance testing, to help reduce the potential impact of prolonged break periods on virus transmission in their local community. Strong policy measures such as wearing facemasks, testing and reduced capacity have shown large reductions in infections in campus models^11^. Our findings corroborate the importance of travel restrictions in limiting COVID-19 disease spread^18^. They are also in line with recent guidelines by the Centers for Disease Control and Prevention to ensure full vaccination prior to traveling, and to decrease travel among students who are at increased risk for severe COVID-19^19^. Along with knowledge of current COVID-19 transmission dynamics and understanding of student adherence to public health recommendations^20,21^, this analysis can aid potential risk assessments at universities across the country. For example, it is likely that the decline in infections at the university used for data calibration can be attributed to intervention protocols implemented locally at the university level, complementing other state and local measures^22^. These policies can enable universities to welcome students back to campus with fewer outbreaks and repercussions to surrounding communities^23^. In contrast, lack of intervention can contribute to increased peaks of infection among students two weeks after travel, suggesting secondary spread^17^.

We acknowledge some limitations of our study. The model parameters were estimated based on limited data and published literature as the explicit semester-wide case data on college campuses were not available. Given uncertainty over parameters about SARS-CoV-2 transmission, the upper and lower bounds of the percent reduction may be subjective to the dynamics we chose. However, the scope of the sensitivity analysis of Fig. 2 reflects a likely inclusive range that has appeared on many college campuses. More data regarding student behavior and COVID-19 cases may help improve our predictions. Since predicting student travel behavior, especially during a pandemic, is complicated, we modeled a wide range of potential behaviors. Institutes of higher education can compare knowledge of their student population with the student travel behavior curves to best utilize our results.

In summary, simulation results, based on computational and mathematical modeling, show that student travel does impact transmission dynamics on college campuses, and that adjusting academic calendars to limit student movement can reduce disease burden. Selecting the correct adjustment to simultaneously mitigate risk and provide students with adequate respite should factor in prevalence projections and the likelihood of the student body to follow recommended safety protocols.

## Methods

### Alternative break schedules

The timing and distribution of the four alternative break schedules were based on a sample of twenty-five universities/colleges that implemented an adjusted break schedule in spring 2021. Among the sample (n = 25), spring breaks were either shortened or rescheduled later in the semester (one-break schedule; n = 9), split into two break-periods (two-break schedule; n = 5), split into three break-periods (three-break schedule; n = 7), or split into four break-periods (four-break schedule; n = 4). Within each schedule-break-period, the start-day of the break relative to the first day of class was determined. These start-days were weighed by their respective duration and averaged to provide simulation start-days for each break-period within each alternative schedules. The duration of each break-period was predetermined such that each alternative schedule had a total of nine days off and was therefore comparable.

### Student travel behavior curve

To mimic the student travel volume observed in mobility data that increased as the break duration increased and saturated at some level, a sigmoid function was selected representing the relationship between break-duration and percent of students that travel (Fig. 1, top). SafeGraph mobility data, based on anonymized aggregated location-based data, was analyzed (from January through November 2020) for a college campus to determine student movement behaviors when presented with breaks of various lengths. The number of cellular devices outside of the college’s county during Thanksgiving break 2020 (a five-day break-period) was approximately three times larger than the number of devices during Labor Day weekend 2020 (a three-day break-period). We maintained this relationship by shifting our sigmoid function such that the proportion of students who travel given a five-day break is three times the amount given a three-day break. To cover a widely varying potential scenario, the final functions were obtained by scaling this shifted sigmoid function such that the percent of students who travel given a nine-day break ranged from 5 to 30%.

### Infections while traveling

Every simulation is associated with a student travel behavior curve, a mean daily contact rate, and a mean travel destination prevalence rate. For a given break-period, the percent of students who travel was determined from the student travel behavior curve. It was assumed that no student would travel for leisure if given a break-period of two days or less. The proportion of students infected while traveling was determined from Monte Carlo (MC) simulations. MC simulations output the proportion of traveling students infected given a break-duration, mean destination prevalence, and mean daily contacts. This percent was applied to the number of students traveling to determine total infections which were distributed uniformly over the break-period.

### Monte Carlo simulation

Monte Carlo simulations were used to estimate the percent of students infected while traveling. The number of daily contacts per student was drawn from a Poisson distribution. The probability of contact with an infectious individual was based on the assumed mean prevalence rate. The probability of viral transmission during contact with an infectious individual was 0.366% and 0.270% from a symptomatic and asymptomatic person, respectively. These transmission rates were based on the estimated $\beta$_i_ and $\beta$_a_ parameters in the SEIAR model. For each break-duration, mean destination prevalence rate, and mean daily contact parameter, 500 simulations of 1000 students were run totaling 105,000 simulations. The mean percent of infected students given a combination of break-duration, mean prevalence, and mean daily contact parameter was applied to traveling students to determine the number of traveling students that returned infected.

### Transmission dynamics model

**Figure 3:**
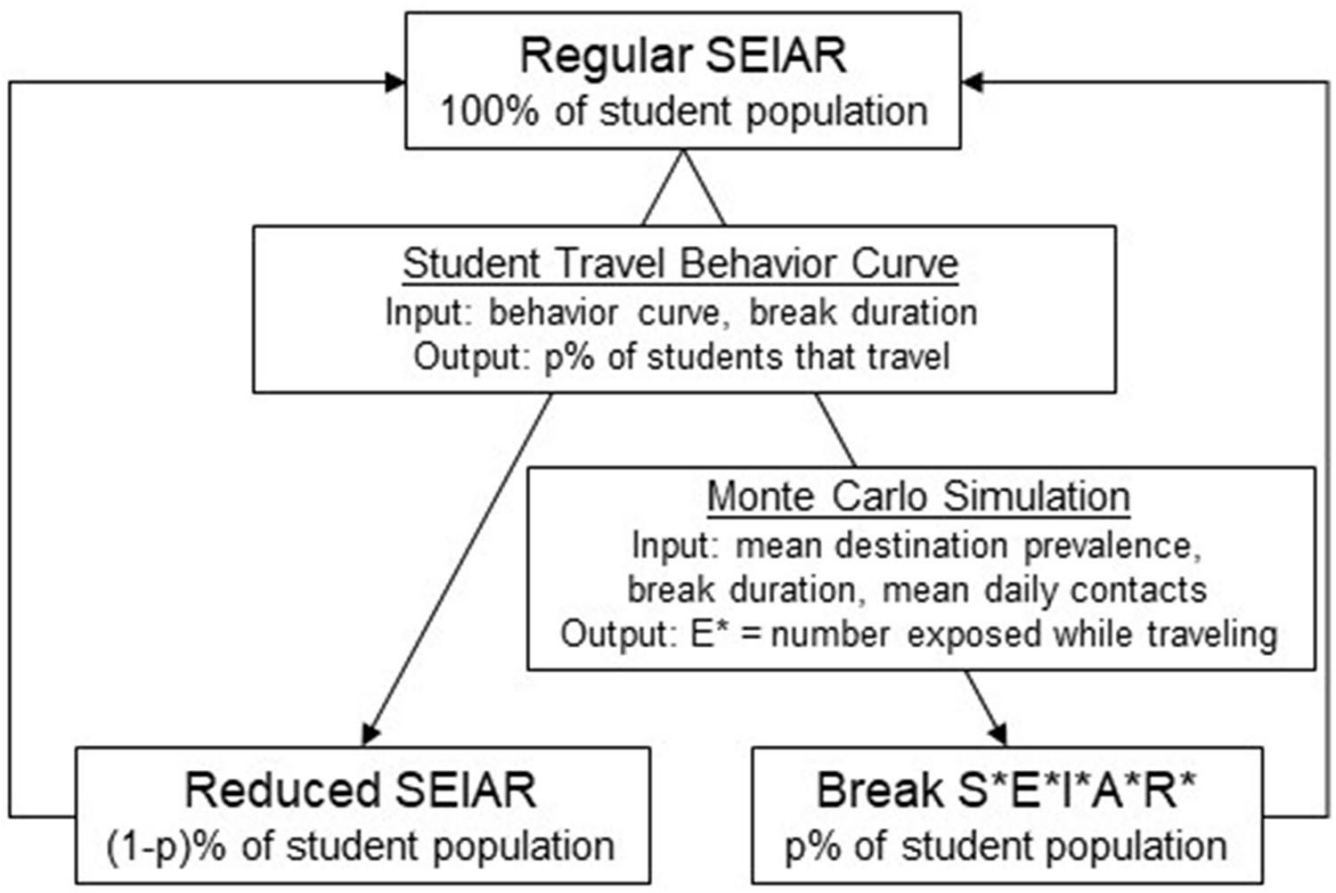
Computational model workflow. During regular-period, transmission dynamics at universities were simulated by a susceptible-exposed-infectious-asymptomatic-recovered model (Regular SEIAR). Student behavior scenarios determined how many students traveled (p%) given a specific number of days off. At the beginning of a break, traveling students left the Regular SEIAR compartments and followed the Break S*E*I*A*R* model, where Monte Carlo simulations determined the number of traveling students infected under a mean destination prevalence and mean daily contact scenario. During break-period, students [(1 − p)%] who did not travel followed SEIAR model (Reduced SEIAR). At the end of the break, traveling students return to the Regular SEIAR compartments until the next scheduled break or the end of the semester.

The schematic diagram of our model simulations is presented in Fig. 3. The model was used to simulate transmission dynamics over the course of a 122-day semester. Specifically, the simulations were performed during regular-period and break-period sequentially using the following models.

#### Regular-period model simulation

A susceptible-exposed-infectious-asymptomatic-recovered (SEIAR) compartmental model was used to simulate transmission dynamics during the regular-period of the semester. The SEIAR model assigned students into mutually exclusive compartments: susceptible (those susceptible to the virus), exposed (those with the virus but not yet capable of transmitting it), infectious (symptomatic persons capable of transmitting the virus to susceptible persons), asymptomatic (asymptomatic persons also capable of transmitting the virus to susceptible persons) and recovered (those who had the virus before and are assumed immune). We assumed a closed student population with no deaths. Transmission occurred during contact between infectious/asymptomatic and susceptible compartments. After successful transmission (determined by transmission parameters $\beta$_i_ and $\beta$_a_), students transitioned from susceptible to exposed and remained in exposed for $1/\sigma$ days on average. Among those in the exposed class, ($1 - \theta$) proportion became symptomatic and the remaining $\theta$ proportion remained asymptomatic. After $1/\lambda$ days on average, students in symptomatic/asymptomatic classes became recovered where they remained immune throughout the rest of the simulation.

The following equations were used to simulate transmission dynamics:

$$\frac{dS\left( t \right)}{{dt}} = - \frac{{\beta_{i} I\left( t \right) + \beta_{a} A\left( t \right)}}{N}* S\left( t \right),$$

$$\frac{dE\left( t \right)}{{dt}} = \frac{{\beta_{i} I\left( t \right) + \beta_{a} A\left( t \right)}}{N}* S\left( t \right) - \sigma E\left( t \right),$$

$$\frac{dI\left( t \right)}{{dt}} = \left( {1 - \theta } \right)\sigma E\left( t \right) - \lambda I\left( t \right),$$

$$\frac{dA\left( t \right)}{{dt}} = \theta \sigma E\left( t \right) - \lambda A\left( t \right),$$

$$\frac{dR\left( t \right)}{{dt}} = \lambda \left( {I\left( t \right) + A\left( t \right)} \right),$$

where $S\left( t \right), E\left( t \right), I\left( t \right), A\left( t \right),$ and $R\left( t \right)$ are the size of the susceptible, exposed, infectious, asymptomatic, and recovered compartments at time $t$, respectively. $\beta_{i }$ and $\beta_{a}$ are the transmission rates from infectious and asymptomatic individuals, respectively, to susceptible individuals. As mentioned above, $1/\sigma$, $1/\lambda$, and $\theta$ represent the incubation period, the infectious period, and the proportion of infections that are asymptomatic, respectively.

#### Break-period model simulation

At the beginning of the break-periods, $p$ proportion of students were selected from all compartments (except infectious) to leave the local compartments for travel. During break-period, $\left( {1 - p} \right)$ proportion of students, who did not travel, followed SEIAR dynamics (referred as *Reduced SEIAR* in Fig. 3). The students, who travelled ($p$ proportion), also continued to transition between compartments (referred as Break S*E*I*A*R* in Fig. 3), however, total transitions to the exposed compartment were predetermined and distributed uniformly over break-periods (MC simulation). The following equations were used to simulate transmission in students who were traveling.

$$\frac{{dS^{*} \left( t \right)}}{dt} = - \left( {N_{T} *a} \right) / D,$$

$$\frac{{dE^{*} \left( t \right)}}{dt} = \left( {N_{T} *a} \right) / D - \sigma E^{*} \left( t \right).$$

The equations for $I^{*} , A^{*} ,$ and $R^{*}$ are similar to the equations for $I, A,$ and $R$ in *SEIAR* model, respectively. Here, $N_{T}$ is the size of traveling student population (determined from student travel behavior curve), $a$ is the percent of traveling students who became exposed over duration of entire break (estimated from Monte Carlo simulations), and $D$ is the duration of break (days).

Upon return from travel after each break-period, students return to their current compartment in the *Regular SEIAR* model.

### SEIAR parameters

COVID-19 dashboards from eight large universities were surveyed and revealed a common trend of a large surge of COVID-19 cases in the first three-weeks of the Fall 2020 semester followed by a steady decline throughout the semester. Transmission parameters between symptomatic and susceptible individuals, $\beta_{i}$, and the initial size of the exposed compartment, were calibrated using two months of publicly available incidence data from a large public university to mimic this trend. We assume that our $\beta_{i}$ estimate incorporated overall effects of implemented contact reduction strategies and therefore such strategies were not explicitly modeled. The value of $\beta_{i}$ use in our base-case computation is 0.0366 per day per individual.

Remaining model parameters were taken from the literature and were as follows: 5-day incubation period ($\sigma$ = 1/5 per day)^24^, 14-day infectious period ($\lambda$ = 1/14 per day)^25^, 40% of infections were asymptomatic ($\theta$ = 0.4)^26^, 75% relative risk of infection from an asymptomatic individual relative to a symptomatic individual ($\beta_{a}$ = 0.75 $\beta_{i}$)^26^. Initial population size of the S, E, I, A, and R compartments were 4767, 232, 1, 0, and 0, respectively.
